# Ab Initio Derived Classical Force Field for Molecular Dynamics Simulations of ZnO Surfaces in Biological Environment

**DOI:** 10.1021/acs.jpca.3c00424

**Published:** 2023-06-14

**Authors:** Marzieh Saeedimasine, Fredrik Grote, Alexander P. Lyubartsev

**Affiliations:** Department of Materials and Environmental Chemistry, Stockholm University, Stockholm SE-106 91, Sweden

## Abstract

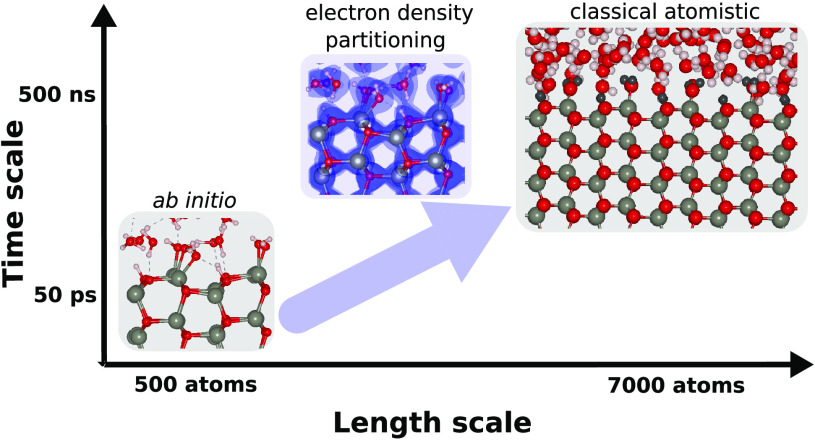

Zinc oxide nanostructures are used in an ever increasing line of applications in technology and biomedical fields. This requires a detailed understanding of the phenomena that occur at the surface particularly in aqueous environments and in contact with biomolecules. In this work, we used ab initio molecular dynamics (AIMD) simulations to determine structural details of ZnO surfaces in water and to develop a general and transferable classical force field for hydrated ZnO surfaces. AIMD simulations show that water molecules dissociate near unmodified ZnO surfaces, forming hydroxyl groups at about 65% of the surface Zn atoms and protonating 3-coordinated surface oxygen atoms, while the rest of the surface Zn atoms bind molecularly adsorbed waters. Several force field atom types for ZnO surface atoms were identified by analysis of the specific connectivities of atoms. The analysis of the electron density was then used to determine partial charges and Lennard-Jones parameters for the identified force field atom types. The obtained force field was validated by comparison with AIMD results and with available experimental data on adsorption and immersion enthalpies, as well as adsorption free energies of several amino acids in methanol. The developed force field can be used for modeling of ZnO in aqueous and other fluid environments and in interaction with biomolecules.

## Introduction

Zinc oxide nanostructures are highly interesting materials with a broad range of applications in bionanotechnology including cosmetics, sunscreens, pigments, and antibiotics.^1,2^ Due to the use of ZnO in different biomedical and technology fields, the toxic hazard associated with it is a topic of great interest in nanotoxicology.^3^ While pH-dependent dissolution of ZnO nanoparticles in cells has been known as a major toxicity mechanism,^4^ the hazards of ZnO via inhalation, skin toxicity, and molecular mechanism of cytoplasmic membrane damage induced by ZnO are still unclear.^5^ A detailed understanding of the ZnO–water interface and its interaction with biomolecules at the atomistic level is therefore very important.

ZnO has a hexagonal wurtzite-type crystalline structure in its normal form where the (101̅0) and (12̅10) faces together account for about 80% of the total surface area and determine the adsorption properties.^6^ The interaction of nanomaterial surfaces with the biological environment is dominated by interaction with water. It was previously shown that for metal oxides with different shapes the first water layer can be strongly bound to the nanomaterial surface^7−9^ and affect considerably the adsorption of biomolecules to the surface.^10^ Therefore, it is important to obtain a detailed understanding of the hydration structure and water reactivity at the ZnO–water interface before describing ZnO–biomaterial interactions.

Experimentally the ZnO–water interface can be studied using a number of techniques. Calorimetry can provide important information about energies and adsorption thermodynamics by measurement of adsorption and immersion enthalpy.^11,12^ Furthermore, surface structure can be studied with FTIR spectroscopy^13^ and NMR^14^ can inform about both the structure and dynamics at the ZnO nanosurfaces. Although these experimental techniques bring together valuable information about the ZnO–water interface, they cannot provide detailed atomistic insights. Here computer simulations can play an essential role.

Several computational methods have been employed to describe water adsorption on ZnO surfaces including density functional theory (DFT) calculations,^15^ ab initio molecular dynamics (AIMD) simulation,^16^ reactive force field (ReaxFF),^17^ density functional based tight binding (DFTB),^18^ and machine learning.^19^ Köppen et al.^16^ compared some of them in modeling of ZnO surfaces interacting with water at different coverage levels. While these methods produce similar results at low water coverage, stronger discrepancies among them are observed for structuring of water layers in contact with the ZnO surface at full hydration. The discrepancies can be traced to the complexity of water adsorption on ZnO surfaces which combines molecularly and dissociatively adsorbed water, or to limitations of each method in the description of ZnO–water interfaces. AIMD simulations can describe accurately the dynamics of dissociative water (chemically bound hydroxyl groups) and molecularly bound water molecules to ZnO surfaces but is limited to small sized systems. The ReaxFF is capable of describing chemical reactions (breaking and formation of water bound to the surface) in larger systems, but it has many parameters which should be fitted by quantum calculation, and it is less computationally efficient than classical force fields (FFs). There are some classical FFs describing bulk ZnO nanomaterials,^20^ but they were not parametrized for surface properties which are crucial for studying interactions of biomolecules with hydrated ZnO surfaces. In a recent paper,^21^ a classical force field for ZnO surfaces was proposed based on fitting of DFT-computed interaction energies of small molecules with a ZnO surface and it was used to compute the adsorption free energy of several amino acids to ZnO in methanol.

The aim of this work is to develop a general classical force field for hydrated ZnO surfaces which is consistent with ab initio modeling, and which can describe the surface properties of ZnO in aqueous and other solvent environments and in interaction with organic and biomolecules. Biomolecular force fields, including only Lennard-Jones and electrostatic potentials for nonbonded interactions, have proved their robustness and high computational efficiency in simulations of hundred thousands (and even millions) of atoms on a beyond microsecond time scale.^22^ Here we develop a force field which has the same functional form and thus is compatible with available biomolecular force fields. We implement a recently developed methodology to determine classical force field parameters from a representative ensemble of atomistic configurations and electron densities determined in AIMD simulations of fully hydrated nanosurfaces.^23^ We further verify the developed force field by computing adsorption enthalpies of water and binding free energies of several biomolecules to the ZnO surface in water and methanol solutions and compare with existing experimental data.

## Material Models and Methods

### Atomistic Force Field Parametrization from Partitioning of Electron Density

We aim to develop a standard molecular mechanics force field for ZnO which includes Lennard-Jones and electrostatic potentials to describe nonbonded interactions, and harmonic bond and angular interactions for atoms connected by bonds/angles (torsion potentials are not used for interactions within ZnO samples, but they are used as a part of the force field describing biomolecules). More details on the exact functional forms of the force field are given in the Supporting Information. We parametrize the force field based on the analysis of the electron density computed in AIMD simulations.^24^ Partitioning of the electron density between atoms provides partial atom charges and Lennard-Jones parameters for all atom types in the simulated system except for nonbound water for which the standard TiP3P model^25^ was used. This method was recently applied for parametrization of the force field for aqueous TiO_2_ surfaces.^23^ A brief recapitulation of the method is given below.

As a first step, AIMD simulations of ZnO slabs fully hydrated by water are carried out to generate an ensemble of equilibrium system snapshots representing possible arrangements of atoms. Then for each snapshot, the electron density *n*(*r*) is analyzed by the Density Derived Electrostatic and Chemical (DDEC6) partitioning method developed by Manz and Limas^24^ to extract net atomic charges (NACs), cubed atomic moments (CAMs), and bond order (BO) for bonds as follows:
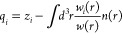
1
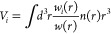
2
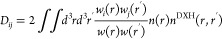
3Here *n*(*r*) is the total electron density, *w*(*r*) = *∑*_*i*_*w*_*i*_(*r*), where *w*_*i*_ is spherical weight assigned to atom *i* by the partitioning method, and *n*^DXH^(*r*, *r*′) is a normalized probability distribution which quantifies the dressed exchange hole.^26^ NACs (*q*_*i*_) computed and averaged over all atoms of a given type provide values of the partial charge for each atom type. The CAM (*V*_*i*_) corresponds to an effective atomic volume which is according to Tkatchenko–Scheffler theory^27^ related to the dispersion coefficient (*B*) in the Lennard-Jones potential:
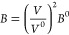
4where *V*^0^ and *B*^0^ are the reference volume and dispersion coefficient for a free atom in vacuum. *B*^0^ was reported for all atoms in rows 1–6 of the periodic table by Gould and Bucko,^28^ while *V*^0^ was determined by DFT computation for a single atom (eq 2). The repulsive parameter of the Lennard-Jones potential (*A*) was determined by a simple scaling relationship:
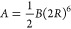
5where *R* is the effective van der Waals radius of the atom in the molecule that was estimated from the CAM and the radius of the free atom in a vacuum (*R*^0^) by^27^
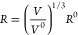
6

Note that since eq 5 is an empirical relation, it provides only preliminary estimation of the repulsive Lennard-Jones parameter. Further fine-tuning of this parameter was made by fitting the first maxima of the relevant radial distribution functions (RDF) to the ones determined from AIMD simulation.

BO (*D*_*ij*_) determines the amount of shared electron density between atoms *i* and *j* and can thus be used to indicate whether these atoms are connected by covalent bond. In this study, we initially defined the local connectivity of atoms identifying the force field atom types by a simple distance cutoff criteria, and then complemented with BO analysis verifying that the bonds defined in the force field indeed correspond to chemical bonds between the atoms.

For bonded force field parameters, we obtained bond distance and angle distributions from AIMD trajectories and fitted them to normal distribution functions to get equilibrium distances (*b*_0_), angles values (θ_0_), and force constants (*k*) for each bond and angle type.

### Training Systems

**Figure 1:**
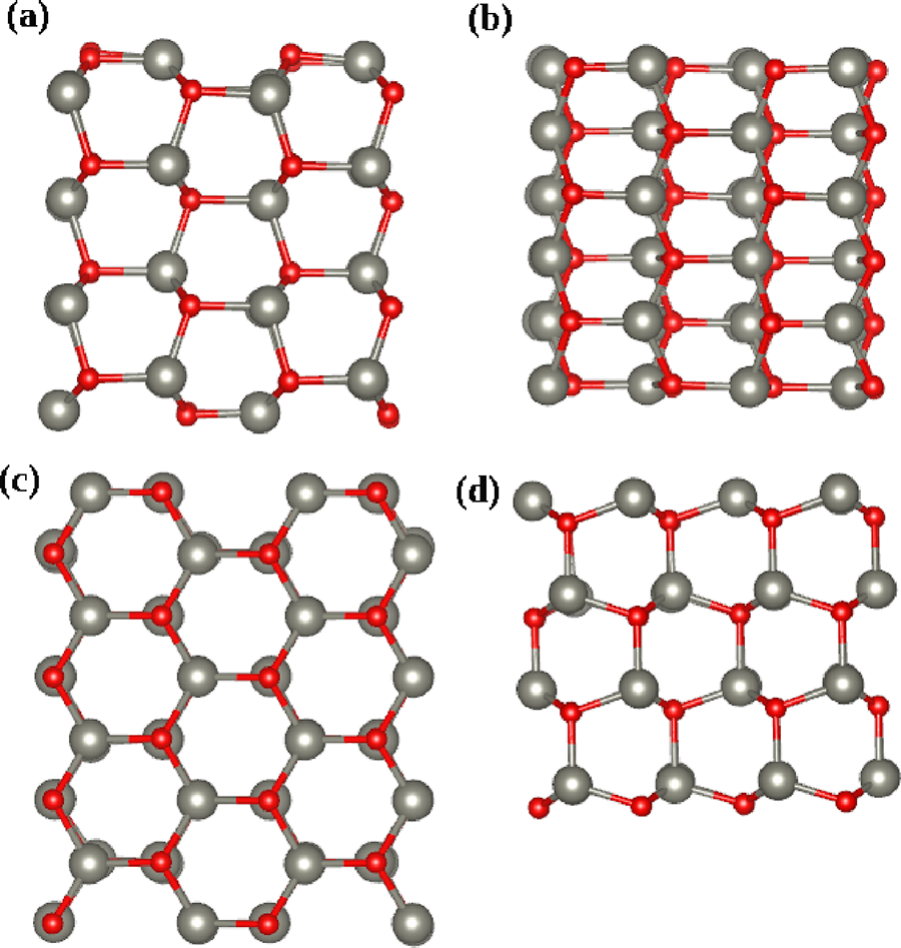
Schematic representation of (a) side and (b) top views of (101̅0) and (c) side and (d) top views of (12̅10) ZnO simulated surfaces.

Two ZnO planar surfaces determined by (101̅0) and (12̅10) Miller indices were simulated in this work. 2D-periodic slab models were constructed by replicating the ZnO wurtzite unit cell and cutting along the corresponding plane using Vesta software.^29^ We used (2 × 3) and (2 × 2) supercells for (101̅0) and (12̅10) surfaces, and 4 layers of ZnO were used as slab thickness which corresponds to (1.06 × 1.01 × 0.98 nm^3^) and (1.14 × 1.06 × 1.31 nm^3^) slab volume (see Figure 1). In both cases, the surface normal was aligned with the *z*-direction of the simulation box. The slabs were fully hydrated with an approximately 1 nm layer of water on both sides to minimize periodic interactions along the *z* direction. Table 1 contains information about the simulated systems.

**Table 1:** Details of ZnO Slab Systems Used in the AIMD Simulations

surface	unit cells	box size (nm)	*N*_slab_	*N*_water_
(101̅0)	2 × 3 × 2	1.06 × 1.01 × 3.62	96	89
(12̅10)	2 × 2 × 4	1.14 × 1.06 × 3.62	128	91

### AIMD Simulation

Born–Oppenheimer AIMD simulations were carried out on the DFT level using the Gaussian and Plane Wave (GPW) method^30^ as implemented in the QUICKSTEP^31^ module of the CP2K package.^32^ The GPW method uses dual plane wave and contracted Gaussian basis sets. We included plane waves with kinetic energy up to a cutoff 300 Ry and used the MOLOPT-DZVP basis set^33^ together with GTH pseudopotentials.^34^ A 20 Ry relative cutoff was used, and the target accuracy for the SCF convergence was set to 5 × 10^–7^. Exchange-correlation was described using the BLYP functional.^35,36^ It is well-known that this exchange-correlation functional does not adequately describe the dispersion energy, and we therefore used Grimme’s D3 dispersion correction.^37^ Full geometry and cell optimization was performed for the (101̅0) and (12̅10) ZnO slabs. Then the surfaces were hydrated with approximately 50 water molecules on both sides and simulated in a NVT ensemble using 50 ps AIMD simulation. The time step to integrate equations of motion was set to 0.5 fs. The last 30 ps of AIMD simulation was used for data production. The system temperature was kept at 300 K using the thermostat of Bussi, Donadio, and Parrinello^38^ with a time constant of 0.1 ps.

### Classical MD Simulations

Classical MD simulations were carried out to fine-tune our predicted force field parameters, and to validate the force field by comparison with experimental data.

To get finally optimized force field parameters, 200 ns NVT MD simulations were performed for the same systems as in AIMD simulations and the final configuration of AIMD was used as the starting geometry. These simulations were used to tune the repulsive parameter of the Lennard-Jones interactions by fitting the first maximum of the RDFs for Zn–O and O–O pair interactions to the results of AIMD simulations.

To validate our finalized force field parameters by experimental data, larger sized ZnO surfaces (3 × 3 × 2 nm slab models) have been created and dressed with bound hydroxyl and water molecules (full details are given in the Results and Discussion). Hydrated ZnO surfaces were equilibrated in a NPT ensemble at a pressure of 1 bar. After that 600 ns production simulations were run in a NVT ensemble to compare thermodynamic properties with available experimental data. The chemical composition of the simulated systems is given in Table S1 of the Supporting Information.

Classical MD simulations in both small and large boxes were carried out using Verlet algorithm with a 2 fs time step. All bonds to hydrogen atoms were constrained by applying the LINCS algorithm.^39^ A V-rescale thermostat^38^ with a relaxation time of 1 ps was used to keep the temperature *T* = 300 K constant. For equilibration of large systems in NPT ensemble semiisotropic Berendsen barostat with a time constant of 2 ps (compressibility of 4.5 × 10^–5^ bar^–1^) was used. In simulations of small systems (of the same size as in the AIMD simulations), cutoff distance was set to 0.44 nm to fit for the box size and neighbor list parameter of 0.46 nm. For large box systems, a 1 nm cutoff distance was employed. Particle-mesh Ewald summation for electrostatic and Lennard-Jones interactions^40^ with grid spacing of 0.12 nm, and with a shift of the real-space part of the potentials at the cutoff distance, was used in all simulations. Note that the use of particle-mesh Ewald for Lennard-Jones interaction makes it possible to use a short cutoff distance while reproducing long-range dispersion interactions. All classical MD simulations have been performed via the Gromacs v.2021 simulation package.^41^

For computation of the binding free energy of biomolecules on ZnO surfaces, advanced sampling metadynamics simulations have been carried out with the PLUMED plug-in v 2.7^42^ to Gromacs v.2021.^41^ Metadynamics simulations, performed in water and methanol solvents, were run for up to 1000 ns simulation time in a NVT ensemble with a constant Gaussian height of 0.001 kJ/mol deposited every 500 steps. The first 50 ns of simulations was excluded from average force computations. The surface separation distance (SSD) is used as a collective variable. It is determined as the *z*-component of the distance between the center of mass of the surface atoms (outermost layer of Zn and O atoms) and center of mass of the adsorbate. The potential of mean force profile was reconstructed from the average force acting on an adsorbent molecule at each SSD, as it was done in our previous work.^43^ The biomolecules and methanol solvent were described by the General Amber Force Field (GAFF) (version 2.11) with parameters generated by running antechamber^44^ via acpype.^45^ The partial charges of amino acids and methanol were computed by the DDEC6^24^ approach for geometries optimized with the Hartree–Fock method and 3-21 G* basis set with polarization functions on heavy atoms.

For all simulated systems by classical MD, the charge of the slab was brought to the nearest integer value by a uniform shift of charges of all surface atoms, and then the total charge of the system was neutralized by adding a suitable number of counterions (Cl^–^ or Na^+^). For binding free energy calculations, additional number of the same ions was added to the system to yield salt concentration at physiological conditions (0.15 M).

## Results and Discussion

We used AIMD simulations to characterize the hydration state of ZnO surfaces and to derive parameters for the atomistic force field. We then validated the obtained force field by calculating some thermodynamic properties using atomistic MD simulations of ZnO in water and methanol solutions.

### ZnO Surface Characterization at Full Hydration

**Figure 2:**
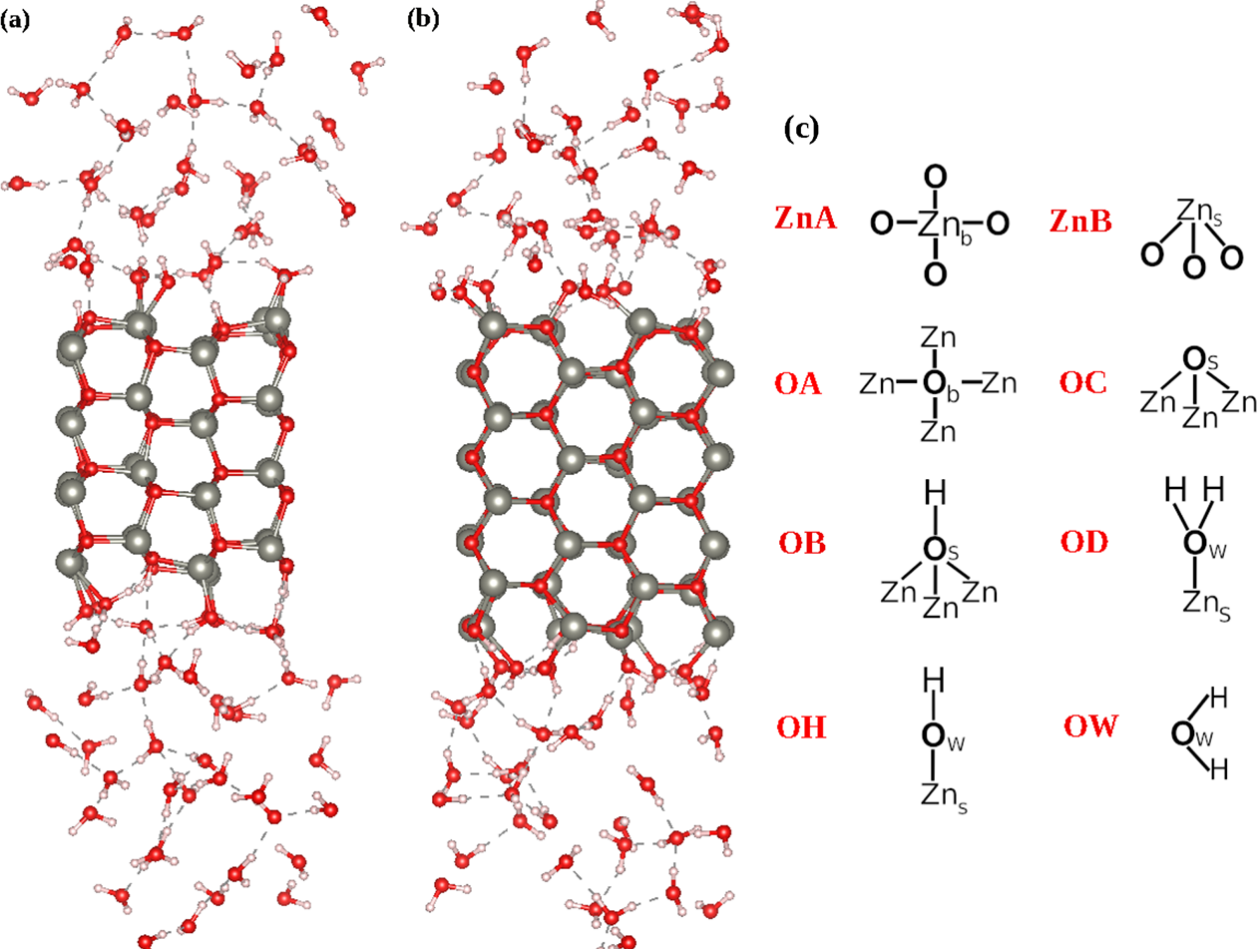
Equilibrated structure of ZnO (101̅0) (a) and ZnO (12̅10) (b) interacting with bulk water at 50 ps of AIMD simulation. (c) Schematic representation of different atom types in ZnO–water simulated systems. The subscripts b, s, and w refer to bulk, surface, and water, respectively.

Visual inspection of the AIMD trajectories showed that the surfaces of ZnO slabs were modified during the simulations with the formation of hydroxyl groups and protonated oxygens, as well as by close coordination of water molecules to the surface Zn atom sites. During these transformations, some water molecules near the surface split, forming a OH group bound to a surface Zn atom, and a proton bound to a neighbor surface O atom site. Water molecules were also observed to be adsorbed molecularly on the surface with a rather short (about 2 Å) distance between the water oxygen and surface Zn atom. The hydroxylation and proton transfer reactivity on ZnO surfaces happens in the first 1–3 ps of the simulation and fluctuates around the equilibrium structure for the rest of the AIMD simulations. The equilibrated structures of ZnO surfaces in water at 50 ps are shown in Figure 2. Further observation showed that the most stable structure on the ZnO (101̅0) surface is formed by a honeycomb network (6-rings) of water molecules that bind to the surface by hydrogen bonds, while a row-like ladder of hydrogen bonded water is more favorable on ZnO (12̅10). Meyer et al.^15^ reported a key–lock structural arrangement for the hydration mechanism of ZnO surfaces which is consistent with our observations.

In order to investigate the presence of various atomic groups at ZnO surfaces, we first identified atom types according to their local connectivity. Each atom type is defined as a unique chemical environment in the first coordination shell around a central atom. Here we defined the chemical environment of each atom by considering other atoms in the first coordination shell determined by the first minimum of the respective RDF. Thus, we used values of 0.25 and 0.13 nm as cutoff distances determining the nearest neighbors of O atoms around Zn and H atoms around O, respectively. We re-evaluate this simple definition of the chemical environment by a cutoff distance using the bond order concept in the discussion below.

We assigned type ZnA to all originally 4-coordinated Zn atoms inside the slabs (bulk), as well as to surface Zn atoms which become 4-coordinated due to attached hydroxyl groups. For Zn surface atoms interacting with molecularly adsorbed water, we assigned type ZnB. For oxygen atoms, we identified six different atom types:

- OA: O–Zn_4_ oxygen atom coordinated by 4 Zn atoms which are found in the bulk material.
- OB: O–Zn_3_H_1_ protonated surface oxygen atom coordinated by 3 Zn atoms.
- OC: O–Zn_3_ surface oxygen atom coordinated by 3 Zn atoms.
- OD: O–Zn_1_H_2_ oxygen of water molecule adsorbed to a surface Zn atom.
- OH: O–Zn_1_H_1_ oxygen of hydroxyl group bound to the surface Zn atom.
- OW: O–H_2_ oxygen of water molecules.

Schematic representations of the identified atom types are shown in Figure 2.

**Figure 3:**
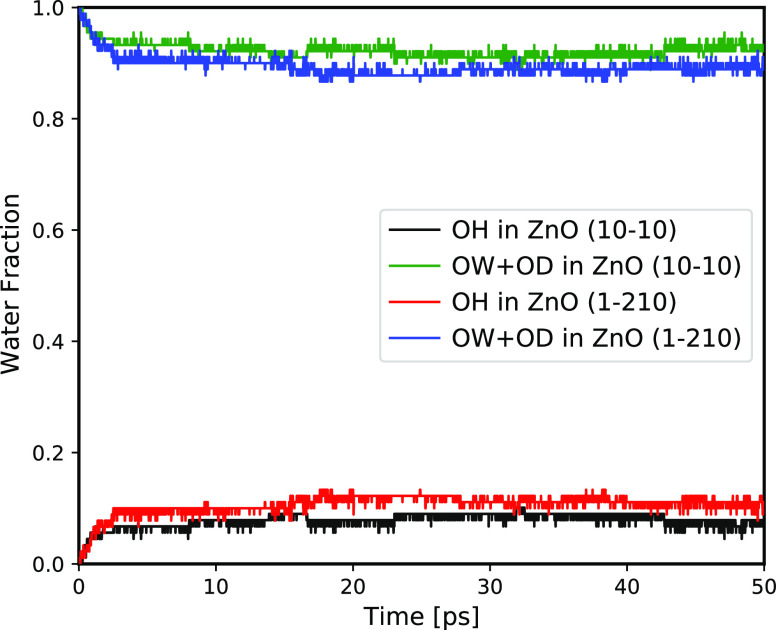
Fraction of dissociated water (OH atom type) and two-coordinated water by hydrogen atoms (OW+OD atom types) as a function of AIMD simulation time in ZnO–water systems.

To quantify water reactivity on ZnO surfaces, we display in Figure 3 the time evolution of the fraction of water (OW + OD) and OH (hydroxyl) oxygen atoms types. At the beginning of AIMD simulation, all water oxygen atoms were two-coordinated by hydrogen atoms (fraction of OW+OD atom type is equal to 1). After several ps of AIMD simulation, as the result of surface reactions, the fraction of water oxygens is decreasing below 1 while oxygens forming hydroxyl groups (type OH) appear. The hydroxylation and proton transfer reactivity on both ZnO surfaces takes 1–3 ps, and after that the fractions of OH and OW atom types fluctuate around the established equilibrium value for the rest of the AIMD simulations.

In order to characterize the ZnO surface modification due to water splitting reactions, we in Figure S1 show the evolution of the number of all surface-related oxygen atom types with respect to the number of surface Zn interaction sites (12 and 16 in ZnO (101̅0) and ZnO (12̅10) simulated systems respectively). The average fraction of these atom types computed during the production part of AIMD simulations is given in Table 2. The fractions of different oxygen atom types per surface Zn atoms are similar for both ZnO surfaces. On average, 0.62 and 0.63 fractions of Zn surface atom sites become hydroxylated (OH atom type) on ZnO (101̅0) and ZnO (12̅10) and the rest of the Zn atom sites are hydrated by molecularly adsorbed water (OD population is 0.38 and 0.37 for ZnO (101̅0) and ZnO (12̅10), respectively). So, we can conclude that the surface Zn interaction sites become either hydroxylated or molecularly hydrated at an approximately 2:1 hydroxyl-water ratio. Surface oxygen atom types (OB and OC) are divided in similar proportions: 64% and 65% of surface O atoms (on ZnO (101̅0) and ZnO (12̅10) systems) become protonated (OB type) while the rest (36% and 35% respectively) retain OC type. One can note that the fraction of protonated oxygen sites exceeds slightly the number of hydroxyl groups by 2%, which indicates that the number of adsorbed protons due to water dissociation is slightly higher than the number of adsorbed hydroxyl groups, and that some small amount of OH^–^ ions remained in water phase.

**Table 2:** Population Analysis of Different Surface Oxygen Atom Types in ZnO–Water Systems^a^

type	label	ZnO(101̅0)	ZnO(12̅10)
OB	O–Zn_3_H_1_	0.64 ± 0.05	0.65 ± 0.05
OC	O–Zn_3_	0.36 ± 0.05	0.35 ± 0.05
OD	O–Zn_1_H_2_	0.38 ± 0.07	0.37 ± 0.07
OH	O–Zn_1_H_1_	0.62 ± 0.07	0.63 ± 0.05

a The values are averaged over last 30 ps of AIMD simulations.

Meyer and co-workers^15,46^ using both STM experiment and quantum mechanical calculation found that at one water monolayer coverage the most stable interface of ZnO (101̅0) is formed when every second water molecule becomes dissociated (1:1 hydroxyl-water ratio). Our result shows that at full hydration the hydroxylation of the surface is twice as common as that of molecularly adsorbed water for both ZnO surfaces. The higher level of hydroxylation can be due to the increased possibility of hydrogen bonding with the bulk water compared to the first water monolayer. Raymand et al.^47,48^ reported that the level of hydroxylation ranges between 50% and 80% for different ZnO–water interfaces in simulations with ReaxFF MD simulation which is consistent with our results. In another simulation study, Quaranta et al.^19^ employed a high-dimensional neural-network potential based on density functional theory data to elucidate the structural and dynamical properties of the ZnO(101̅0)–water surface. They showed hydroxylation and proton transfer on the ZnO surface within a few ps of simulation time and reported surface hydroxylation of 71% and 29% molecularly bond water to the ZnO(101̅0) which is also consistent with our observations.

### Assigning Classical Force Field Parameters

**Figure 4:**
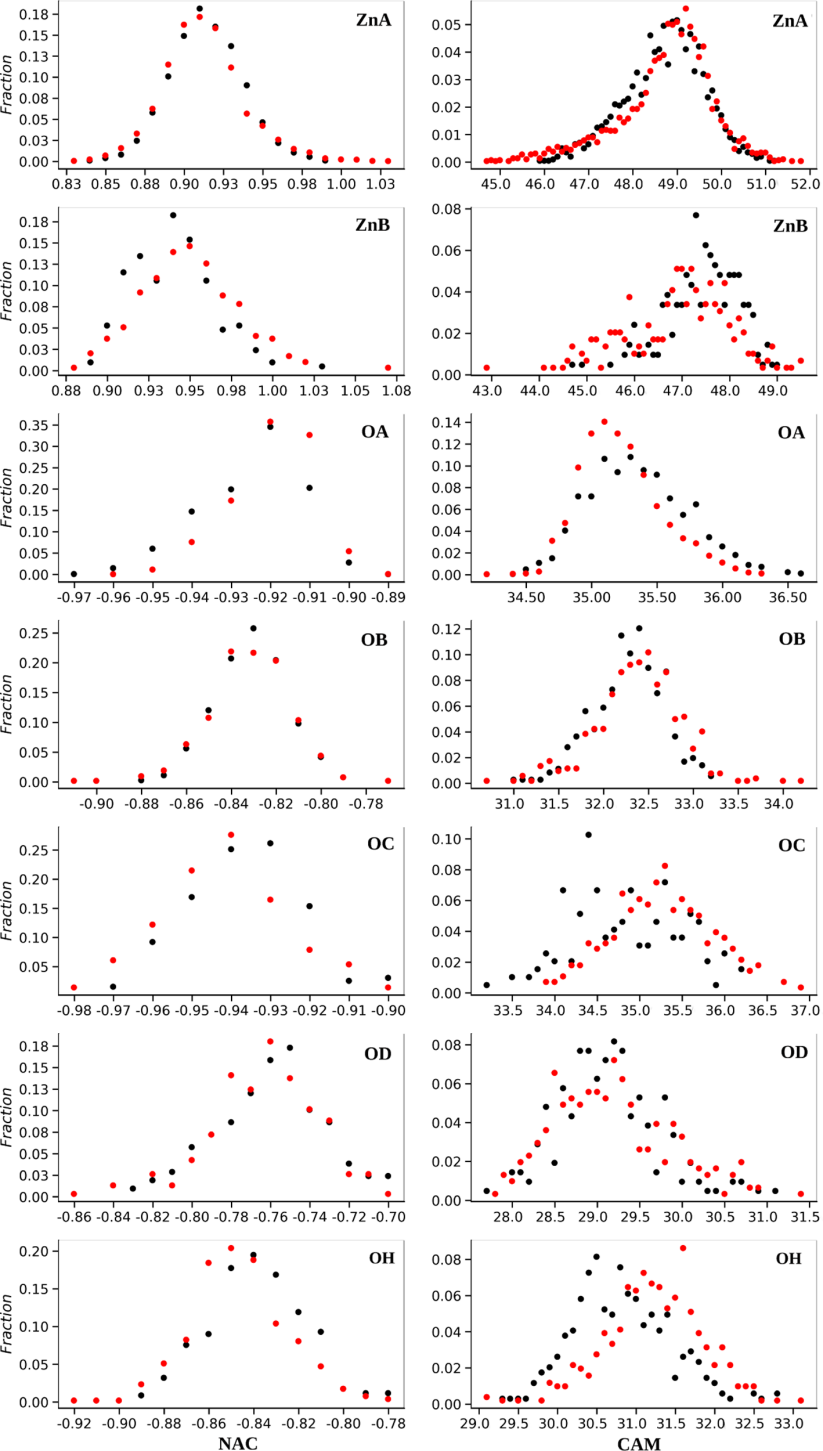
Histogram of the NAC (left) and CAM (right) for different atom types of ZnO(101̅0)–water in black and ZnO(12̅10)–water in red.

Classical force field parameters were obtained from analysis of the electron density as described in the Material Models and Methods section. AIMD simulations provided a set of snapshots representing thermal fluctuations of the atoms in the system. We extracted snapshots each 0.5 ps of the production part of the simulation trajectories with an electron density resolution of 0.1 Å. The DDEC6^24^ method was used to determine NAC and CAM using Chargemol v 3.4 software^49^ for a collection of 120 snapshots from the two simulated surfaces. Histograms of NAC and CAM for different atom types are shown in Figure 4.

We assigned the average vaĺue of NAC as partial charge for each atom type with the exception of oxygens of free water molecules (OW) and all hydrogens which we kept equal to the charges in the TIP3P water model (0.417 *e* for hydrogen and −0.834 *e* for water oxygen). For each atom type, the NAC and CAM distribution and average value are very similar for both surfaces (see Figure 4) which justifies our choice of force field atom types based on chemical connectivity which is transferable over different surfaces. The finalized values of partial charges for all atom types are listed in Table 3. By assigning these charges, the bulk ZnO becomes neutral, while the surface gets a slightly positive surface charge which is consistent with the experimentally measured positive zeta potential of ZnO at neutral pH.^50,51^ We discuss surface charge and zeta potential in more detail in the text below.

**Table 3:** Finalized Nonbonded Force Field Parameters for ZnO–Water Interfaces in the NB Model Predicted by Partitioning of Ab Initio Electron Density and Tuned with Classical MD Simulation

type	label	charge (*e*)	σ (nm)	ϵ (kJmol^–1^)
ZnA	Zn–O_4_	0.918	0.318	1.241
ZnB	Zn–O_3_	0.949	0.150	1.241
OA	O–Zn_4_	–0.918	0.343	0.372
OB	O–Zn_3_H_1_	–0.827	0.323	0.437
OC	O–Zn_3_	–0.934	0.307	0.709
OH	O–Zn_1_H_1_	–0.838	0.313	0.476
OW	O–H_2_	–0.834	0.315	0.636
HA	H–O_1_	0.417	0.000	0.000

The average value of CAM for each atom type was used to determine the dispersion *B* parameter in the Lennard-Jones potential using eq 4. We computed *V*^0^ values for free Zn and O atoms in vacuum as 78.99 *a*_0_^3^ and 23.52 *a*_0_^3^ using the same settings for the basis set, plane wave cutoff, etc. as used in the AIMD simulations of hydrated ZnO surfaces. *B*^0^ parameters were taken from Gould and Bucko^28^ as equal to 276 Ha *a*_0_^6^ and 16.7 Ha *a*_0_^6^ for Zn and O atoms, respectively. The repulsive Lennard-Jones parameter (*A*) was initially estimated by considering the effective van der Waals radii 0.210 and 0.176 nm for Zn and O atoms (eqs 5 and 6). The obtained Lennard-Jones parameters in this way are listed in Table S2. These parameters were then tuned in classical MD simulation of a ZnO–water system by matching to the RDFs obtained in AIMD simulations. In order to keep consistency with the TIP3P water model and GAFF parameters for other molecules, we assumed zero Lennard-Jones parameters for hydrogen, and for water oxygen (OW) Lennard-Jones parameters were kept corresponding to TIP3P water.

For covalent bonding and angular interactions, we used harmonic potentials with parameters initially determined from the distribution of interatomic distances and angles for each bond/angle type in AIMD simulation and fitted to normal distribution functions. The mean and standard deviation values were used to calculate equilibrium bond distance (*b*_0_) and force constant values (*k*). The obtained bonded FF parameters are listed in Table S3.

The bond order analysis has been used to determine whether atom coordination initially determined by a distance criteria corresponds to covalent chemical bonds, and particularly determine the type of binding for molecularly bound water. The average bond order value for a proton to the surface oxygen atom (OB–HA) is 0.75, and such protons are modeled as covalently bonded to the surface. The average BOs for hydroxyl to Zn surface atoms (ZnA–OH) and ZnA–OA bonds are 0.65 and 0.5, respectively; these bonds are also modeled with a bonded potential in the force field. For BO between surface Zn atoms and oxygen of molecularly bound water (ZnA–OD), we get a 0.45 average value. While this relatively high BO value indicates partially covalent character of binding of molecularly adsorbed water, we observed also a relatively high exchange rate of such water molecules on the time scale of AIMD simulations, as illustrated by Figure S1. We therefore considered two modeling strategies: in one, the ZnA–OD bond is described as covalent bonded interaction, and in the other it is described as a strong nonbonded interaction presented by a Lennard-Jones potential with specially assigned parameters. We named these models BOND and NB in this paper, and in the following we discuss the suitability of these models in description of ZnO–water surface properties.

In the BOND model, all Zn surface atoms have attached either molecularly bound water or hydroxyl, and they all are assigned as ZnA atom type (4-coordinated Zn). Both hydroxyl and molecularly adsorbed water are bound to the Zn surface atom by harmonic bonds with respective parameters. In the NB model, Zn surface atoms interacting with molecularly adsorbed water are considered as 3-coordinated and are parametrized separately as ZnB atom type. To bring water molecules close to the surface, a short σ distance of 0.150 nm was assigned for the Lennard-Jones parameter of ZnB atom type.

**Figure 5:**
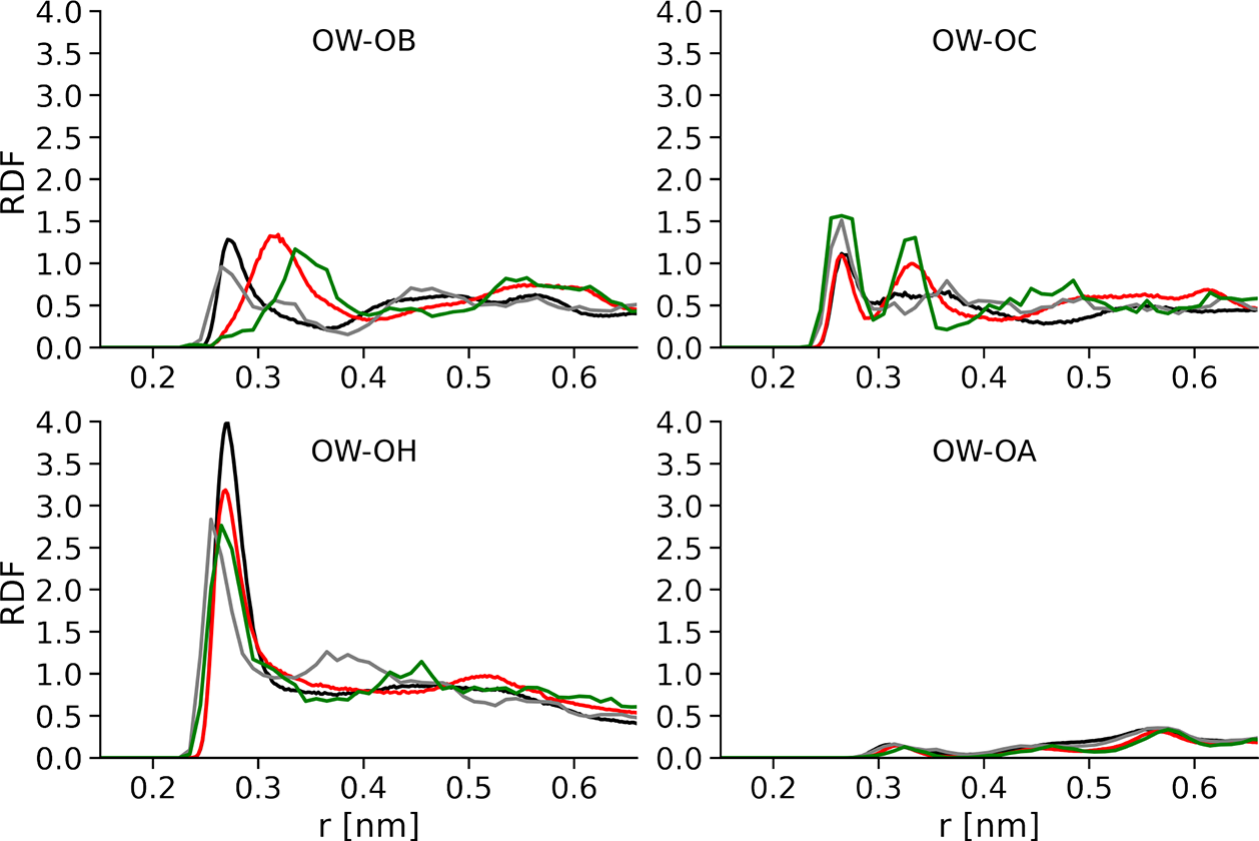
RDFs between different surface O atoms and water oxygens in classical MD simulation compared to AIMDs in a NB model of ZnO–water. ZnO(101̅0) is colored in black, and the corresponding RDF from AIMD is shown in gray. ZnO(12̅10) is colored red, and the corresponding RDF from AIMD is shown in green.

**Figure 6:**
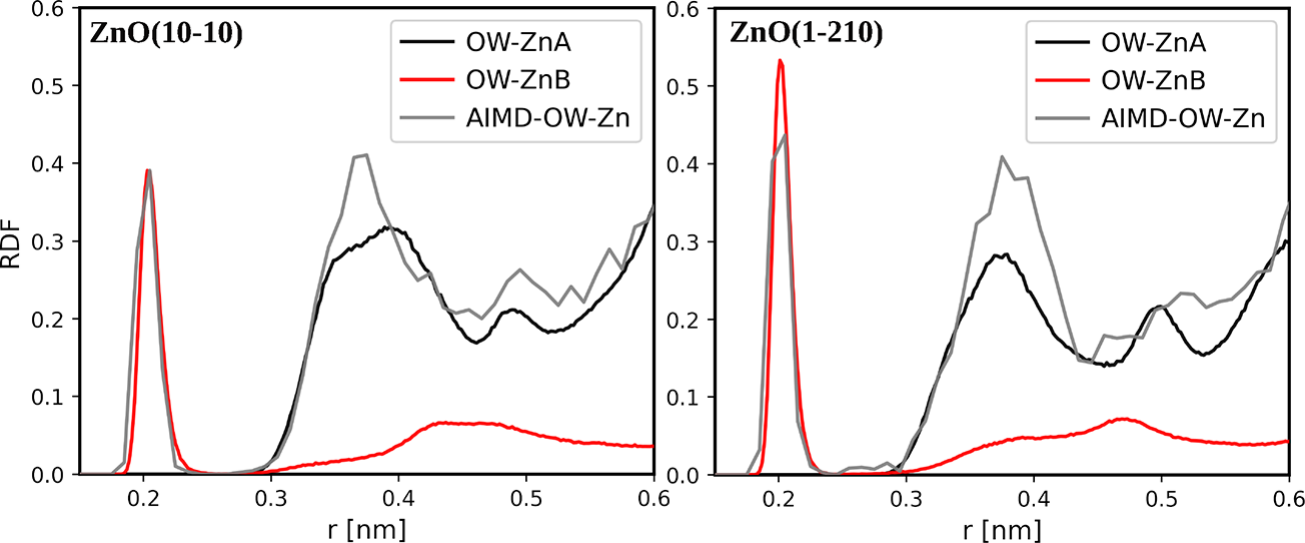
RDFs of Zn atoms (ZnA or ZnB) interacting with water oxygens (OW) in a classical MD simulation of NB model ZnO–water. The corresponding Zn–OW RDF from AIMD is shown in gray.

To fine-tune FF parameters, we apply preliminary obtained values (Tables S2 and S3) and run classical MD simulation in NVT ensemble for the same system as the one in which AIMD simulations were carried out. We varied repulsive parameter *A* of the Lennard-Jones interactions while keeping parameter *B* to fit the first maximum of the respective RDF to the result of AIMD simulations. Also bonded FF parameters were tuned during this comparison, aiming to reproduce average bond length and its variance observed in AIMD simulations. For bonded interactions, the fitted RDFs compared to AIMDs are shown in Figures S2 and S3. For fitted nonbonded parameters of the NB model, a comparison of RDFs between different surface O atoms and water oxygens is shown in Figure 5 while RDFs between surface Zn atoms and water oxygens are shown in Figure 6. For the BOND model, a comparison of RDFs between surface O atoms and water oxygens is shown in Figure S4.

A comparison of classical force field RDFs with AIMD results shows that the BOND model in general reproduces better the water structure around the ZnO surface compared to the NB model. In particular, it is difficult to reproduce the coordination of molecularly bound water obtained by AIMD simulations within the NB model, in which molecularly bound water is presented by the standard TIP3P model and interacts with the ZnO surface only by Lennard-Jones and electrostatic interactions. On the other hand, the BOND model excludes the possibility of dissociation of such water and its eventual substitution by other (nonwater) molecules, which might be important, e.g., in studies of biomolecular adsorption.

**Figure 7:**
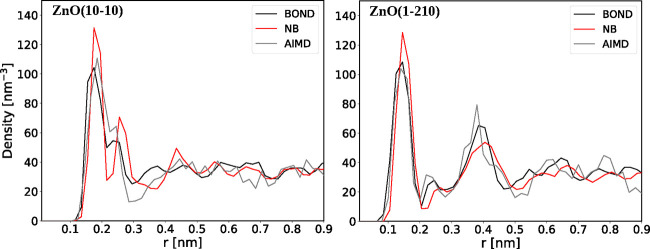
Water number density distributions as a function of *z*-distance from the ZnO surface.

We calculated also water number density distributions as a function of *z*-distance from the ZnO surface (Figure 7). The density distributions for both the BOND and NB models are in good agreement with AIMD results.

The finalized bonded and nonbonded FF parameters for different atom types in the NB model are listed in Tables 3 and 4, and in Tables S4 and S5 for the BOND model of ZnO. An archive of developed FF parameters in Gromacs format is provided as a part of Supporting Information.

**Table 4:** Finalized Bonded Force Field Parameters for ZnO–Water Interfaces in the NB Model Predicted by Partitioning of Ab Initio Electron Density and Tuned with Classical MD Simulation

type	*b*_0_ (nm)	*k* (kJ mol^–1^nm^–2^)
ZnA–OA	0.180	100000.0
ZnA–OB	0.200	100000.0
ZnA–OC	0.175	100000.0
ZnA–OH	0.180	100000.0
ZnB–OA	0.180	100000.0
ZnB–OB	0.185	100000.0
ZnB–OC	0.175	100000.0
OB–HA	0.100	40000.0
OH–HA	0.098	40000.0

type	θ_0_ (deg)	*k*(kJ mol^–1^rad^–2^)
ZnA–OB–HA	104.0	628.0
ZnA–OH–HA	114.0	500.0

### Surface Charge Model

Setting up a realistic surface model is another important question for classical MD simulations of hydrated metal oxide surfaces. Chemical bonds cannot be broken or formed in classical MD; that is why the amount of hydroxylated and protonated groups needs to be set in advance before simulation starts. As it was discussed above (see Table 2), our AIMD simulations showed that about 65% of surface Zn atoms are hydroxylated, a similar fraction of surface oxygens is protonated, and about 35% of surface Zn atoms attach molecularly bound water. To ensure that this distribution of the surface groups is reasonable, it is instructive to compare the surface charge determined by the force field partial charges with experimental data on the zeta potential. ZnO at neutral pH in water solution shows a positive zeta potential of 20–35 mV^50,51^ which corresponds to a 0.2–0.4 *e*/nm^2^ charge density on the ZnO surface at physiological salt concentrations^52^ that should be considered on modeling of ZnO surfaces.

In the BOND model of ZnO, all Zn atoms are assigned ZnA atom type with 0.918 *e* charge. Here, 66% of the surface sites of ZnO(101̅0) was modified by hydroxyl and protonated oxygen groups, which results in 0.293 *e*/nm^2^ surface charge. ZnO(12̅10) was dressed with 63% of OH and protonated oxygen groups which resulted in 0.318 *e*/nm^2^ surface charge. For the NB model, we used a 0.949 *e* charge for ZnB atom type based on NAC analysis (see Figure 4 for ZnB atom type), which is a bit larger than the ZnA charge (0.918 *e*) and results in surface charges of the NB model of ZnO(101̅0) and ZnO(12̅10) of 0.377 *e*/nm^2^ and 0.395 *e*/nm^2^, respectively. These surface charge values are well in line with experimental estimations of the surface charge of ZnO at neutral pH in water solution from zeta potential measurements.^50,51^

Since the zeta potential and subsequently the surface charge of ZnO are solvent dependent, it is always possible to correct the amount of bound OH and protonated oxygen groups on the surface of ZnO to be in line with experimental zeta potential values for the given experimental conditions including solvent, pH, and ion concentration. For example, ZnO shows negative zeta potential in methanol (about −30 mV) .^53^ In this case, we can correct the number of bound OH and protonated oxygen groups on the surface of ZnO models to reach the corresponding negative surface charge in methanol. In simulations of ZnO in methanol described below, the fraction of OH atom type per surface Zn atoms was increased to 75%–80% and the fraction of protonated oxygen decreased to 50%–54%. Thus, our developed FF for ZnO can be transferred for MD simulations in other solvents, pH, and ionic conditions by adjusting the number of surface groups.

### ZnO Force Field Validation

We have carried out a series of simulations to validate how well the developed force field reproduces thermodynamic properties of ZnO surfaces by comparing results from simulations with experimental data. We calculated water adsorption enthalpy, immersion enthalpy, as well as binding affinities of small amino acids on ZnO surfaces in water and methanol solvents. Larger sized (101̅0) and (12̅10) ZnO surfaces (3 × 3 × 2 nm) have been used for these classical MD simulations. The surfaces were modified by setting hydroxyl groups and protonated oxygens at a specified fraction of the surface sites (65% as in AIMD simulations if not said other value) while the rest of the surface sites had molecularly bound water in the BOND model or were left free in the NB model.

#### Water Adsorption Enthalpy

We have computed the water adsorption enthalpy on ZnO surfaces and compared with available experimental data.^11,54^ The differential heat of adsorption is calculated as the enthalpy change due to the small increase of the number of water molecules on the ZnO surface from *n*_1_ to *n*_2_ as follows:
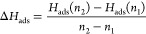
7

**Figure 8:**
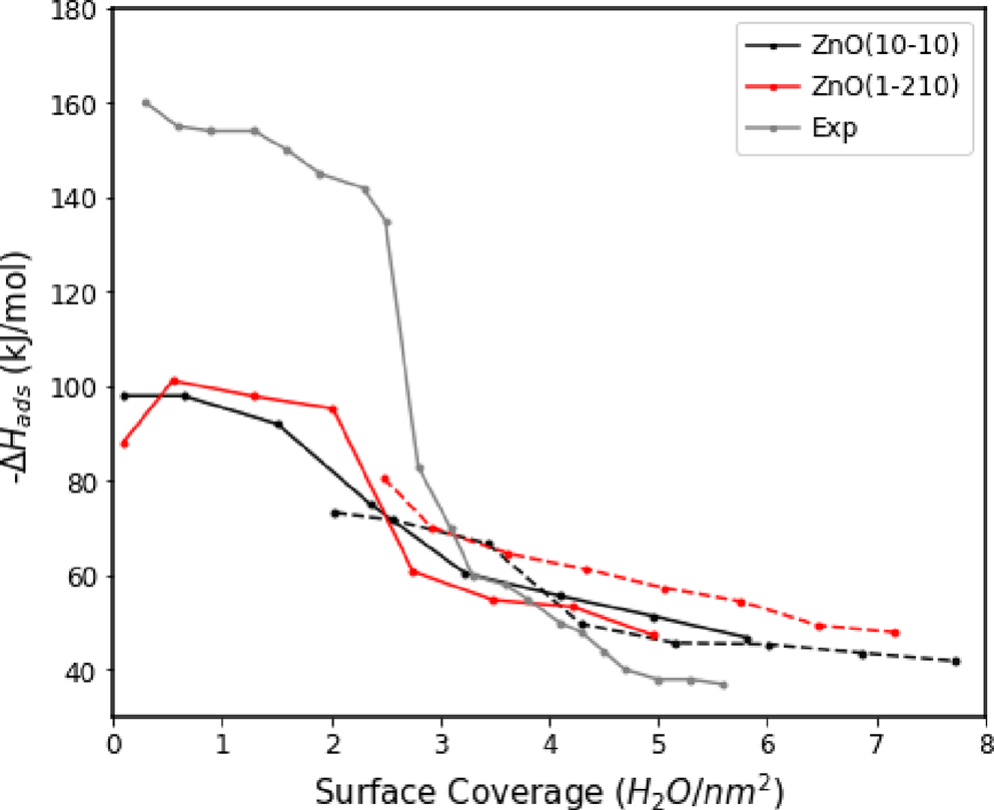
Differential heat of water adsorption on ZnO(101̅0) and ZnO(101̅0) surfaces using classical MD simulation of ZnO models compared to experiment. NB ZnO models are indicated by solid lines, and BOND models are shown by dashed lines.

Thirty different MD simulations have been conducted with an increasing number of water molecules on the (101̅0) and (12̅10) ZnO surfaces considering both BOND and NB models. Differential enthalpy of water adsorption as a function of surface coverage (determined as a function of  per surface area) is shown in Figure 8 along with experimental calorimetry results on ZnO nanoparticles.^54^ Taking into account that in the BOND model molecularly adsorbed water is always bound, we included it when counting water coverage. The computational error values are all within 1 kJ/mol. Two different adsorption modes can be identified in the experimental differential enthalpy of water adsorption. The first part (from 0 to 3 H_2_O/nm^2^ coverage value) reveals highly exothermic adsorption enthalpies and indicates strong water chemisorption, while the second part (from 3 H_2_O/nm^2^ coverage value) shows less exothermic adsorption enthalpies and strong physisorbed water to the surface. The chemisorbed water can be associated with a high first peak of the water density profile in Figure 7, while physisorbed water corresponds to other features of the density profile at larger distances. The BOND model does not reproduce the strong binding of chemisorbed water since molecularly bound water cannot dissociate, but it reproduces well adsorption of physisorbed water which corresponds to the second hydration layer. The NB model shows an increased adsorption enthalpy for the low coverage, but at a weaker level than experimental results, and demonstrates good agreement for the higher surface coverage above 3 H_2_O/nm^2^.

We have also computed water adsorption enthalpies with lower levels of hydroxyls and protonated oxygens at the ZnO surface and present the results in the Supporting Information (Figure S5). We have noted that, by decreasing the amount of OH groups on the ZnO surface, the chemisorbed part is shifted to the larger surface coverage values (4–5 H_2_O/nm^2^) and it deviates from the experiment curve. Zhang et al.^11^ showed that about 2.4–3.3 H_2_O/nm^2^ remains on the ZnO nanomaterial surfaces even after extreme heat treatment (450 °C degassing) which likely originates from dissociated adsorbed water as hydroxyl groups. By comparing these models with experimental results,^54^ we can conclude that the fraction of OH groups at around 65% suits best the experimental results and is also consistent with results of our AIMD simulations.

#### Water Immersion Enthalpy

In order to further validate our FF parameters, we calculated the heat of immersion (*E*_im_) defined as the enthalpy change when the surface is completely immersed in water at constant temperature and pressure. It can be computed from the average potential energy difference between the surface hydrated in water (*E*_interface_), the surface in vacuum (*E*_surface_), and the bulk water with an identical number of water molecules in the hydrated system (*E*_solvent_) as bellow:
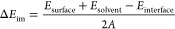
8where *A* is the surface area of the slab. The heat of immersion for NB ZnO models is in the range 0.29–1.16 J m^–2^, while BOND models show lower values of 0.19–0.22 J m^–2^. The experimental values are reported in the range of 0.4–1.4 J m^–2^ for different ZnO nanostructures.^12^ By comparing the water adsorption enthalpy and immersion enthalpy on ZnO model surfaces with experimental data, we can conclude that both the NB and BOND ZnO models can describe thermodynamic properties as well as structural properties (shown by fitting the RDFs) of ZnO.

#### Binding Affinities of Amino Acids to ZnO Surface

To characterize the interaction of biomolecules with ZnO surfaces, we calculated the potentials of mean force (PMF) and binding free energies of several amino acids to ZnO surfaces. We selected three amino acids, alanine (ALA), serine (SER), and histidine (HIS), as representatives of nonpolar, polar, and charged amino acids. Since the zwitterionic form of amino acids is dominant in aqueous media, all simulations were carried out with the amino acids in their zwitterionic form. The NB model of ZnO surfaces has been employed for this calculation because of the possibility of direct interaction between amino acids and Zn atoms. In the NB model, amino acids are able to exchange with molecularly adsorbed water molecules and bind closely to the surface, while in the BOND model the covalent bond defined between molecularly bound water and Zn surface atoms (ZnA–OD covalent bond potential) does not allow amino acids to bind directly to the surface.

Additionally, adsorption simulations of these amino acids were carried out in methanol solvent because of the availability of experimental adsorption data.^21^ Since ZnO shows a negative zeta potential (about −30 mV)^53^ and subsequently a negative surface charge in methanol, we corrected the number of OH and protonated H groups on the surface of ZnO models to reach the corresponding negative surface charge in methanol. By this modification, the fraction of OH atom type per surface Zn atoms increases to 75% and 80% and the fraction of protonated oxygen decreases to 50% and 54% in the NB (101̅0) and (12̅10) ZnO surface models, respectively.

Adsorption profiles of amino acids on ZnO surfaces in water and methanol are shown in Figure S6, and binding free energies are listed in Table 5. Some of the potential of mean force values show more than one minimum, reflecting different binding modes. In Figure S7, we illustrate the interaction of ALA to the ZnO(101̅0) surface in water and in methanol by showing configurations in two main binding modes. In the first binding mode (SSD = 0.42 nm in water), ALA via a carboxyl group binds directly to the surface Zn atom, while in the second binding mode water mediates the interaction of ALA to the surface (SSD = 0.59 nm). By changing the solvent to methanol, ALA gets closer and binds stronger via both carboxyl and amine groups to the surface in the first binding mode (SSD = 0.36 nm in methanol). In the second mode, ALA is stabilized at a larger distance from the surface (0.76 nm) while methanol molecules interact directly with the surface. The second binding mode of ALA in methanol solvent is shifted to larger distances since methanol is a larger molecule than water. A similar behavior has been observed for SER and HIS amino acids.

**Table 5:** Binding free Energy of Amino Acids (Zwitterion form) in kJ mol^–1^ on Different ZnO Model Systems

ZnO models	solvent	ALA	SER	HIS
ZnO(101̅0)	H_2_O	–7.5 ± 3.6	–7.8 ± 2.8	–18.6 ± 5.9
ZnO(12̅10)	H_2_O	–5.6 ± 5.5	–7.7 ± 3.34	–7.4 ± 2.9
ZnO(101̅0)	MEOH	–51.9 ± 10.5	–39.6 ± 11.79	–44.7 ± 5.3
ZnO(12̅10)	MEOH	–29.4 ± 4	–26.2 ± 7.50	–56.3 ± 19.3
ZnO(101̅0)^21,55^	MEOH	–38.9 ± 0.63	–39.96 ± 0.50	–52.43 ± 0.54

The calculated binding free energies show stronger binding of amino acids to ZnO surfaces in methanol compared to water. This increase can be attributed to the negative surface charge of ZnO in methanol. We tested this by computing the binding free energy of SER on ZnO(101̅0) without surface modification in the presence of methanol which resulted in a lower binding free energy value (−15.64 ± 3.8 kJ mol^–1^). Thus, we can conclude that polarization on the ZnO surface is solvent dependent and should be adjusted by the number of bound hydroxyl groups and protons on the ZnO surface. The binding free energies of amino acids in methanol solvent show good agreement with experiment. The error value in the calculation of the binding free energy is relatively large due to the presence of different binding regions on the ZnO surfaces which required longer simulation time to obtain the average interaction of the biomolecule over the surface. From these results, we can conclude that our derived FF model of ZnO gives a realistic description of interaction with biomolecules and can be transferred and adjusted for simulation of ZnO in other solvents.

## Conclusion

In this work, extensive AIMD simulations of ZnO surfaces at full hydration have been carried out in order to gain detailed information about the structure and reactivity at the ZnO–water interface. This information has been quantified to set up a general and transferable classical force field for molecular dynamics simulations of ZnO surfaces in different solvents and in interaction with biomolecules, as well as to set up surface models of ZnO at different conditions. In our AIMD-derived ZnO surface model, in the case of aqueous solution at neutral pH, 65% of Zn surface atoms are hydroxylated and an equal amount of surface oxygen atoms are protonated, which together with ab initio derived partial atom charges provides surface charge and zeta potential consistent with experiment. The rest of the (nonhydroxylated) surface Zn atoms interact strongly with molecularly adsorbed water, which can be considered either permanently bound within the BOND model or dissociable within the NB model, with the latter being provided by the Lennard-Jones potential with a small σ-parameter. The fraction of hydroxylated and protonated atoms at the ZnO surface can be adjusted to provide surface charge at other specific conditions depending on the pH and solvent. The developed classical force field was validated by comparison with AIMD results and further verified by experimental thermodynamic data showing reasonable or moderate agreement. An important point of our force field is that it was derived exclusively from the results of ab initio simulations without using empirical parameters, and it provides a reasonable compromise between accuracy and computational efficiency. We believe that the developed force field can be used for classical large-scale MD simulation of different ZnO surfaces in different solvents as well as in interaction with biomolecules.

## Supporting Information Available

Force field details; chemical characterization of all simulated systems; time evolution of the number of different oxygen atom types; nonbonded and bonded force field parameters; RDFs between different types of O and Zn atoms; finalized nonbonded and bonded force field parameters for ZnO–water interfaces; water adsorption enthalpy obtained in classical MD simulations; PMF profiles of different amino acids; molecular orientation of ALA adsorption on ZnO(101̅0) and ZnO(101̅0) (PDF)
Archive of developed classical force field parameters of ZnO surfaces, ZnO surface models, input files used in the simulations (Gromacs format) (ZIP)
