# Supplementary material for: Ab Initio Derived Classical Force Field for Molecular Dynamics Simulations of ZnO Surfaces in Biological Environment
Source: J Phys Chem A. 2023 Jun 14;127(25):5446–57. doi: 10.1021/acs.jpca.3c00424 (PMC10316393; doi:10.1021/acs.jpca.3c00424)
Supplement: Supplementary file 1 — jp3c00424_si_001.pdf [file jp3c00424_si_001.pdf]

Ab-Initio Derived Classical Force Field for Molecular Dynamics Simulations of ZnO Surfaces in Biological Environment

Supporting Information

Marzieh Saeedimasine, Fredrik Grote, and Alexander P. Lyubartsev*

Department of Materials and Environmental Chemistry, Stockholm University, Stockholm, SE-106 91, Sweden

E-mail: alexander.lyubartsev@mmk.su.se

Force Field details

For ZnO we use force field with non-bonded interactions described by the Lennnard Jones potential and electrostatic interactions calculated for fixed partial charges using Coulombs law. To model bond stretching and angle bending (bonds to hydrogen atoms are constrained) we used standard harmonic potentials. Torsional potentials are not included into ZnO force field. Non-bonded interactions are excluded for pairs of atoms involved in bonded interactions, while for 1-4 Lennard-Jones and electrostatic interactions scaling factors of 0.5 and 0.83 respectively were applied. Lorentz-Berthelot combination rules were used to determine Lennard-Jones parameters for all cross-interactions.

The functional form of the force field is given by the equation:

$$U = \sum_{\text{bonds}} \frac{k_b}{2}(b - b_0)^2 + \sum_{\text{angles}} \frac{k_\theta}{2}(\theta - \theta_0)^2$$

$$+ \sum_{\text{non- bonded}} 4\varepsilon_{ij}\left[\left(\frac{\sigma_{ij}}{r_{ij}}\right)^{12} - \left(\frac{\sigma_{ij}}{r_{ij}}\right)^{6}\right] + \sum_{\text{charges}} \frac{q_i q_j}{4\pi\epsilon_0 r_{ij}}$$

where k_b and k_θ are force constants for bonds and angles respectively; b bond length; b_0 equilibrium bond length; θ angle; θ_0 equilibrium angle; ε_{ij} parameter determining depth of the potential energy minimum in the Lennard Jones potential; σ_{ij} is the Lennard Jones particle radius; r_{ij} distance between non-bonded atoms; q_i atomic charge; ϵ_0 vacuum permittivity.

Table S1: The chemical characterization of all simulated systems in classical MD simulations

**ZnO models**	**Model**	**Solvent**	**Slab**	$\mathbf{N}_{Solvent}$	$\mathbf{N}_{Na}$	$\mathbf{N}_{Cl}$
small systems						
ZnO($10\bar{1}0$)	BOND	H_2O	$Zn_{48}O_{60}H_{24}$	75	-	-
ZnO($1\bar{2}10$)	BOND	H_2O	$Zn_{64}O_{80}H_{32}$	74	-	-
ZnO($10\bar{1}0$)	NB	H_2O	$Zn_{48}O_{56}H_{16}$	79	-	-
ZnO($1\bar{2}10$)	NB	H_2O	$Zn_{64}O_{74}H_{20}$	76	-	-
water adsorption enthalpy calculations						
ZnO($10\bar{1}0$)	BOND	H_2O	$Zn_{864}O_{972}H_{216}$	$0-68$	-	5
ZnO($1\bar{2}10$)	BOND	H_2O	$Zn_{1152}O_{1296}H_{288}$	$0-68$	-	7
ZnO($10\bar{1}0$)	NB	H_2O	$Zn_{864}O_{936}H_{144}$	$0-68$	-	6
ZnO($1\bar{2}10$)	NB	H_2O	$Zn_{1152}O_{1260}H_{180}$	$0-68$	-	8
water immersion enthalpy calculations						
ZnO($10\bar{1}0$)	BOND	H_2O	$Zn_{864}O_{972}H_{216}$	0, 1650	-	5
ZnO($1\bar{2}10$)	BOND	H_2O	$Zn_{1152}O_{1296}H_{288}$	0, 1694	-	7
ZnO($10\bar{1}0$)	NB	H_2O	$Zn_{864}O_{936}H_{144}$	0, 1587	-	6
ZnO($1\bar{2}10$)	NB	H_2O	$Zn_{1152}O_{1260}H_{180}$	0, 1737	-	8
aminoacids adsorption free energies						
ZnO($10\bar{1}0$)	NB	H_2O	$Zn_{864}O_{936}H_{144}$	1669	7	13
ZnO($1\bar{2}10$)	NB	H_2O	$Zn_{1152}O_{1260}H_{180}$	1722	8	16
ZnO($10\bar{1}0$)	NB	MEOH	$Zn_{864}O_{950}H_{144}$	800	13	7
ZnO($1\bar{2}10$)	NB	MEOH	$Zn_{1152}O_{1242}H_{180}$	800	16	8

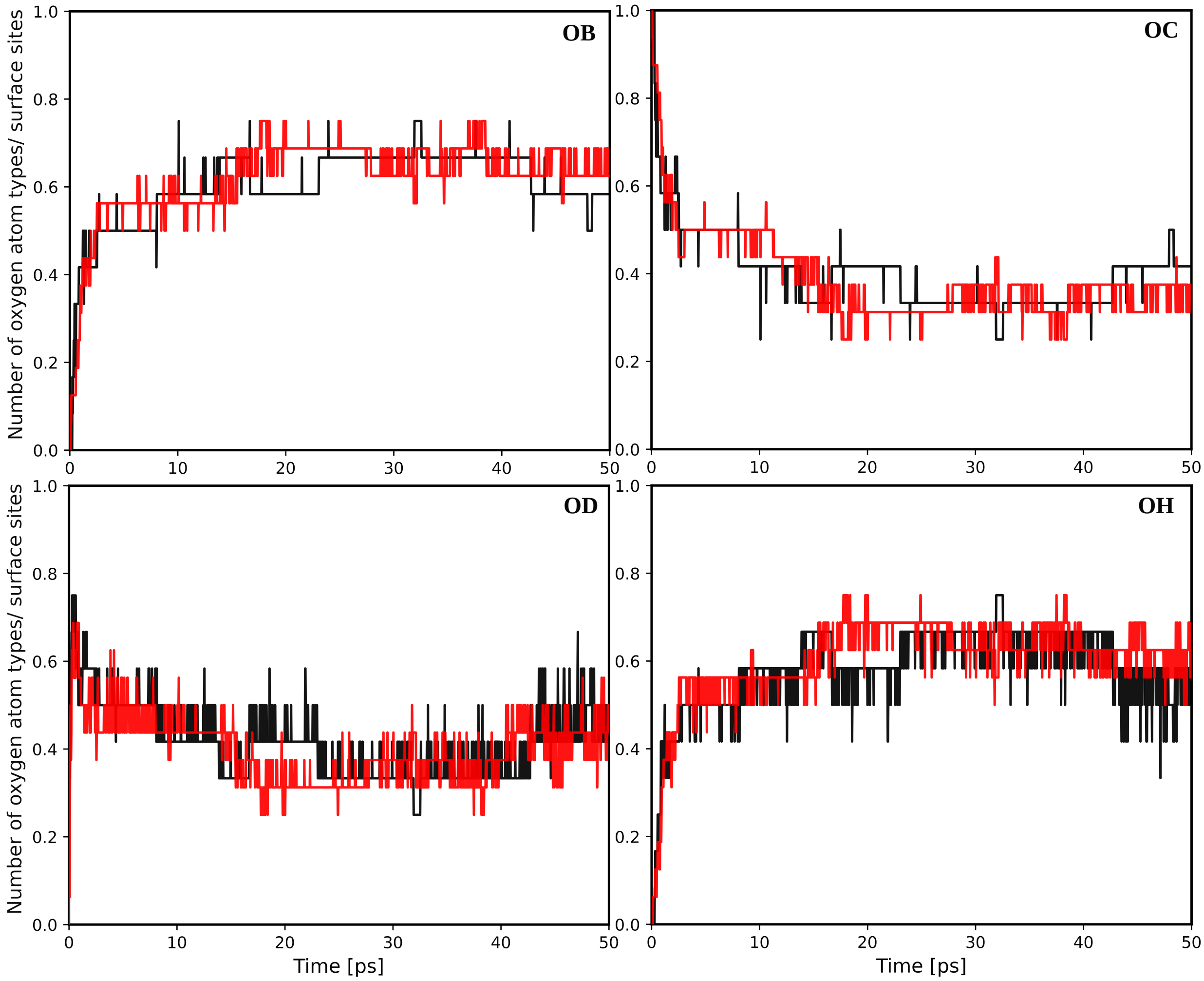

Figure S1: The time evolution of the number of different oxygen atom types (resulted from water dissociation) during AIMD simulations of ZnO $(10\bar{1}0)$ colored in black and ZnO $(1\bar{2}10)$ colored in red. The values are scaled by the number of surface atom sites on each surface. The data were taken from each 0.025 ps of AIMD simulations.

Table S2: Non-bonded force field parameters obtained by partitioning of *ab initio* electron density of ZnO-water systems.

**Type**	**Label**	$\boldsymbol{\sigma}$ **(nm)**	$\boldsymbol{\epsilon}$ **(kJmol^{-1})**
ZnA	$Zn-O_4$	0.318	1.241
OA	$O-Zn_4$	0.343	0.372
OB	$O-Zn_3H_1$	0.333	0.363
OC	$O-Zn_3$	0.341	0.370
OD	$O-Zn_1H_2$	0.322	0.354
OH	$O-Zn_1H_1$	0.328	0.359

Table S3: Bonded force field parameters obtained from AIMD simulation of ZnO-water systems.

**Type**	$\boldsymbol{b_0}$ **(nm)**	$\boldsymbol{k}$ **(kJmol^{-1}nm^{-2})**
ZnA−OA	0.202	25000.0
ZnA−OB	0.209	18000.0
ZnA−OC	0.196	36000.0
ZnA−OD	0.209	18000.0
ZnA−OH	0.196	36000.0
OB−HA	0.100	50000.0
OD−HA	0.101	50000.0
OH−HA	0.099	50000.0
**Type**	$\boldsymbol{\theta_0}$ **(°)**	$\boldsymbol{k}$ **(kJmol^{-1}rad^{-2})**
ZnA−OB−HA	106.0	100.0
ZnA−OD−HA	106.0	100.0
ZnA−OH−HA	107.0	100.0

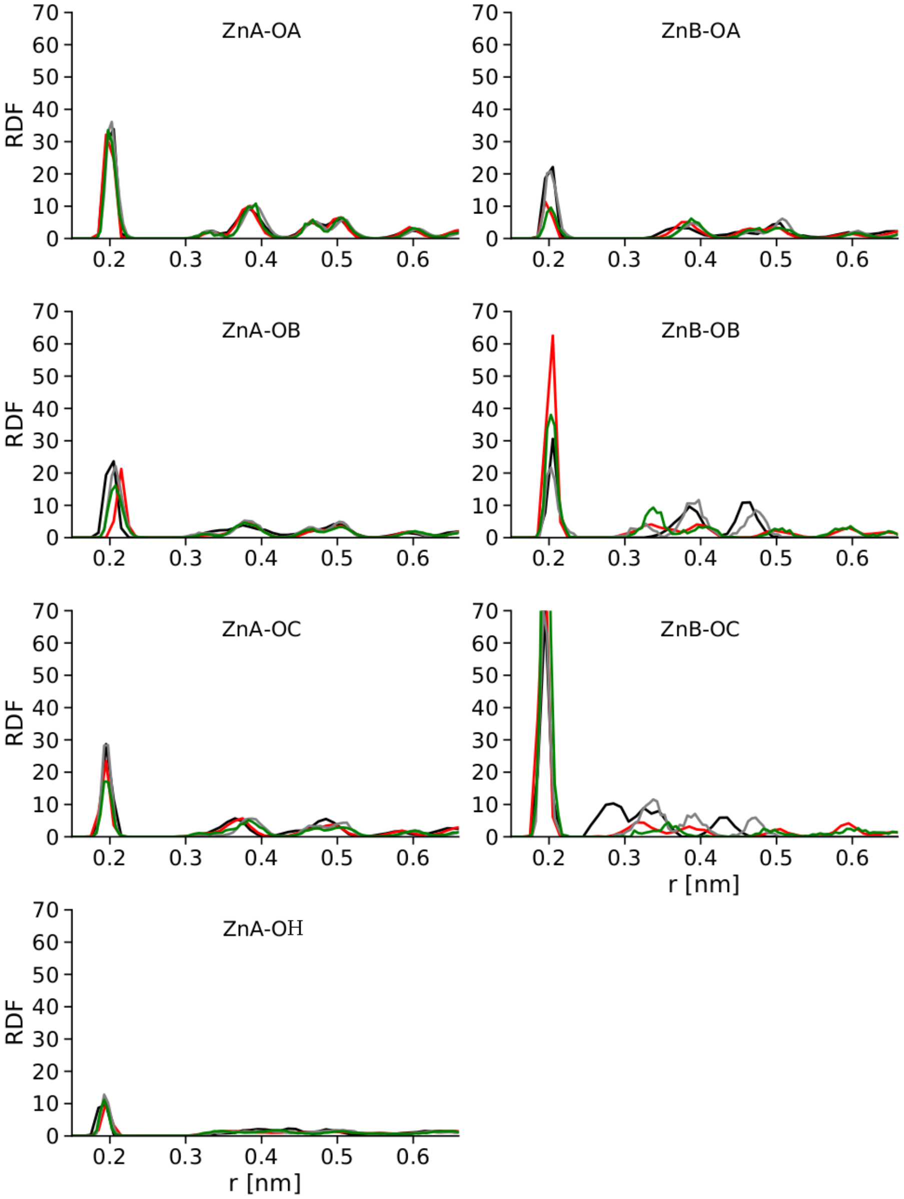

Figure S2: RDFs between different types of O and Zn atoms in classical MD simulation compared to AIMDs in the NB model. ZnO $(10\bar{1}0)$ is colored in black and the corresponding RDF from AIMD is shown in gray. ZnO $(1\bar{2}10)$ is colored red and the corresponding RDF from AIMD is shown in green.

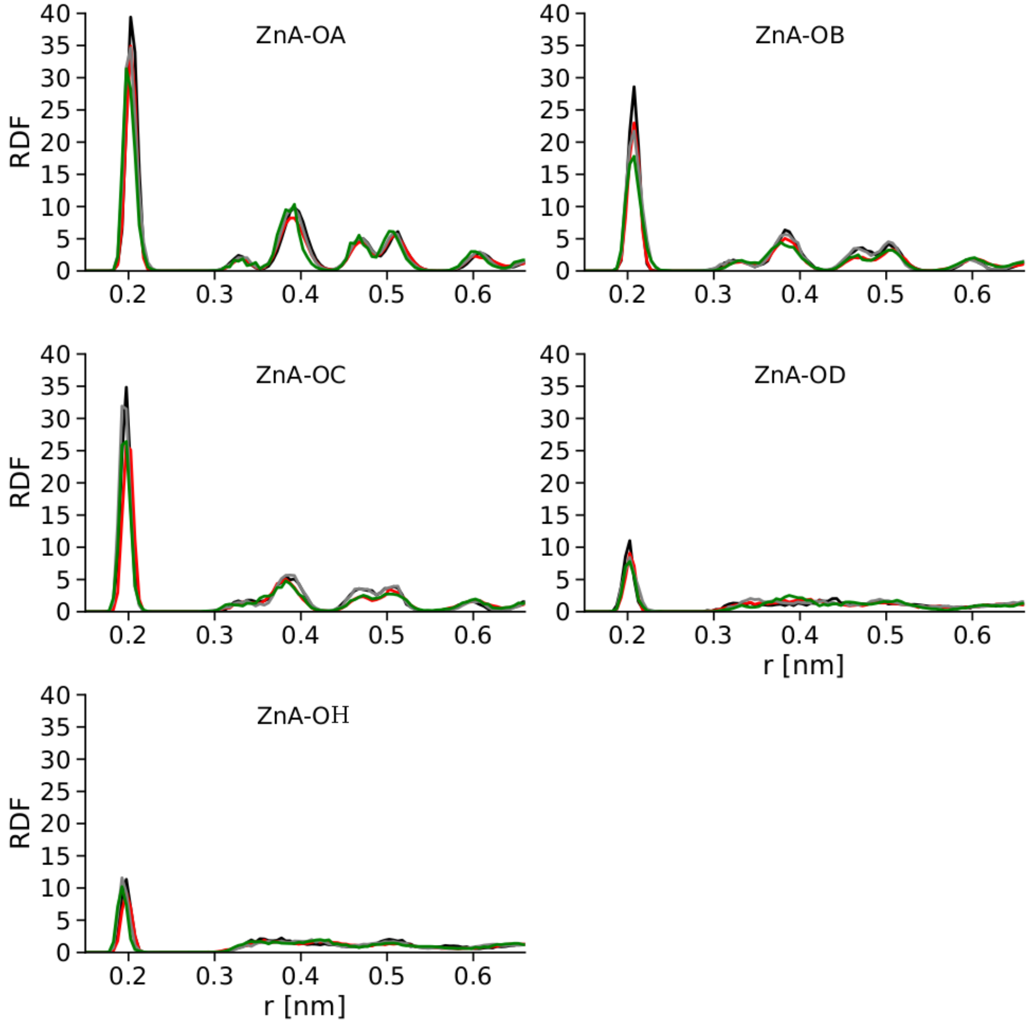

Figure S3: RDFs between different types of O and Zn atoms in classical MD simulation compared to AIMDs in the BOND model. ZnO $(10\bar{1}0)$ is colored in black and the corresponding RDF from AIMD is shown in gray. ZnO $(1\bar{2}10)$ is colored red and the corresponding RDF from AIMD is shown in green.

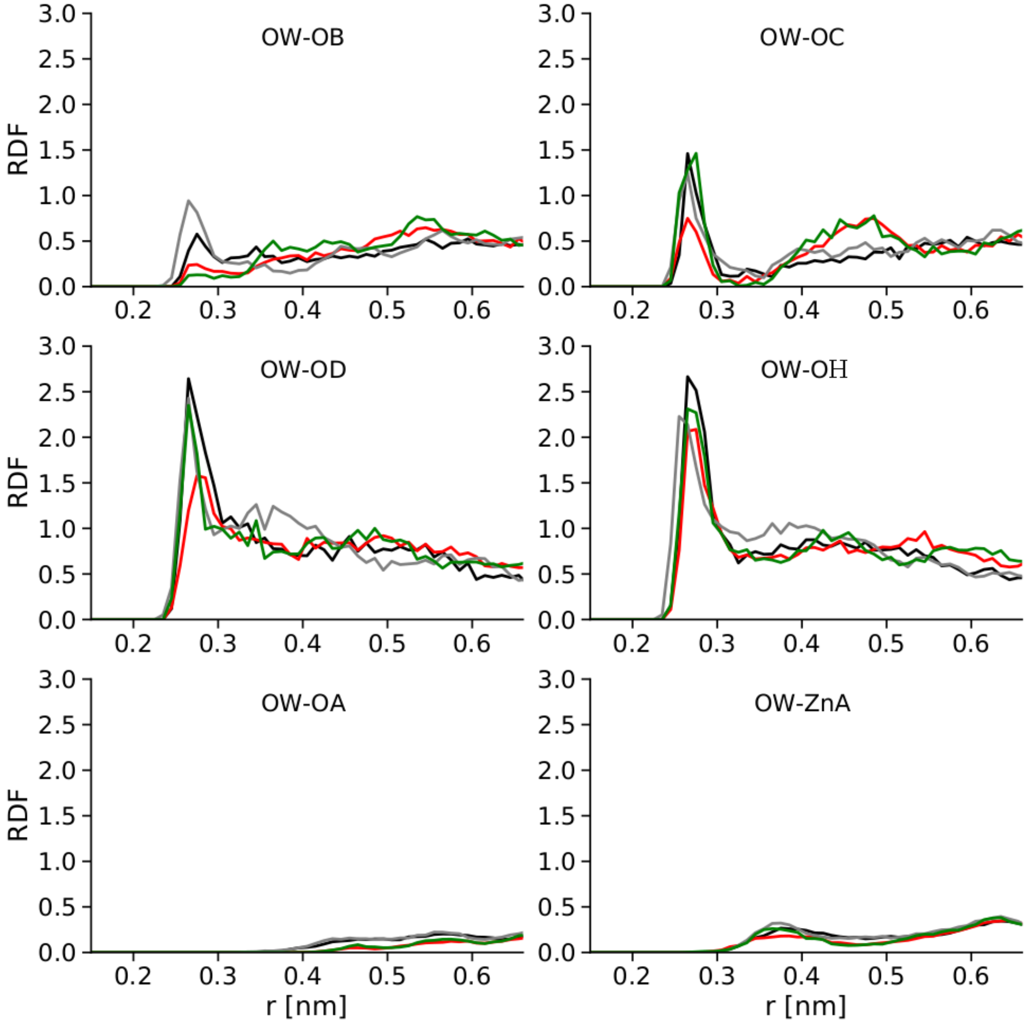

Figure S4: RDFs between different surface O atoms and water oxygen in classical MD simulation compared to AIMDs in BOND model of ZnOs-water. ZnO $(10\bar{1}0)$ is colored in black and the corresponding RDF from AIMD is shown in gray. ZnO $(1\bar{2}10)$ is colored red and the corresponding RDF from AIMD is shown in green.

Table S4: Finalized nonbonded force field parameters for ZnO-water interfaces in BOND model predicted by partitioning of ab initio electron density and tuned with classical MD simulation.

**Type**	**Label**	**Charge (e)**	**σ (nm)**	**ϵ (kJmol^{-1})**
ZnA	Zn$-$O$_4$	0.918	0.318	1.241
OA	O$-$Zn$_4$	-0.918	0.343	0.372
OB	O$-$Zn$_3$H$_1$	-0.827	0.323	0.437
OC	O$-$Zn$_3$	-0.934	0.307	0.709
OD	O$-$Zn$_1$H$_2$	-0.834	0.316	0.396
OH	O$-$Zn$_1$H$_1$	-0.838	0.313	0.476
OW	O$-$H$_2$	-0.834	0.315	0.636
HA	H$_1$	0.417	0.000	0.000

Table S5: Finalized bonded force field parameters for ZnO-water interfaces in BOND model predicted by partitioning of ab initio electron density and tuned with classical MD simulation.

**Type**	**b_0 (nm)**	**k (kJmol^{-1}nm^{-2})**
ZnA$-$OA	0.175	60000.0
ZnA$-$OB	0.176	50000.0
ZnA$-$OC	0.175	70000.0
ZnA$-$OD	0.175	60000.0
ZnA$-$OH	0.175	70000.0
OB$-$HA	0.100	40000.0
OD$-$HA	0.100	40000.0
OH$-$HA	0.098	40000.0
**Type**	**θ_0 ($^\circ$)**	**k (kJmol^{-1}rad^{-2})**
ZnA$-$OB$-$HA	104.0	628.0
ZnA$-$OD$-$HA	114.0	500.0
ZnA$-$OH$-$HA	114.0	500.0

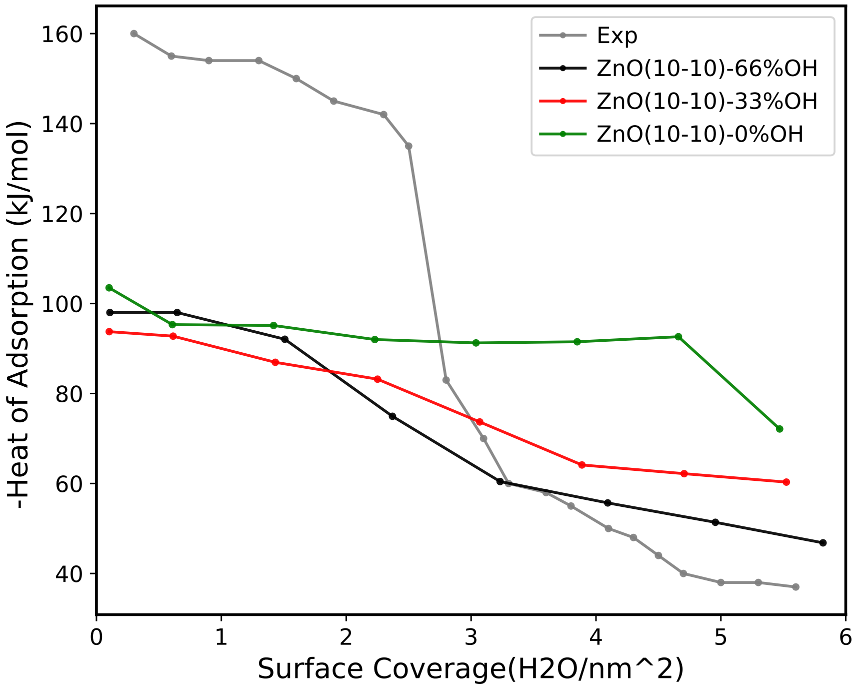

Figure S5: Water adsorption enthalpy obtained in classical MD simulations of NB model of ZnO $(10\bar{1}0)$ surface with different percentage of OH groups.

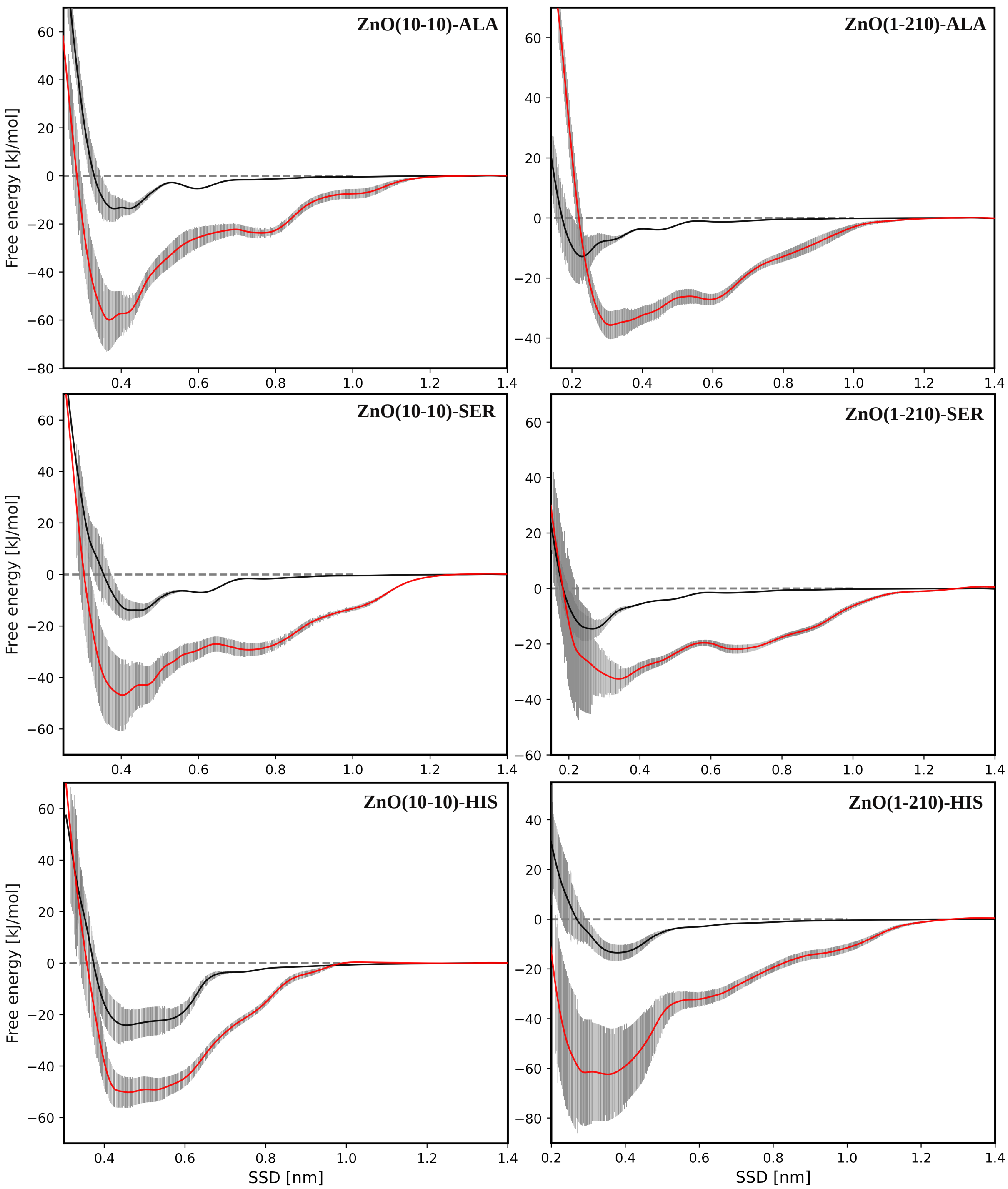

Figure S6: Potential of mean force profiles of different aminoacids on ZnO surfaces in presence of water and methanol solvents, colored in black and red, respectively. The errors are shown in gray.

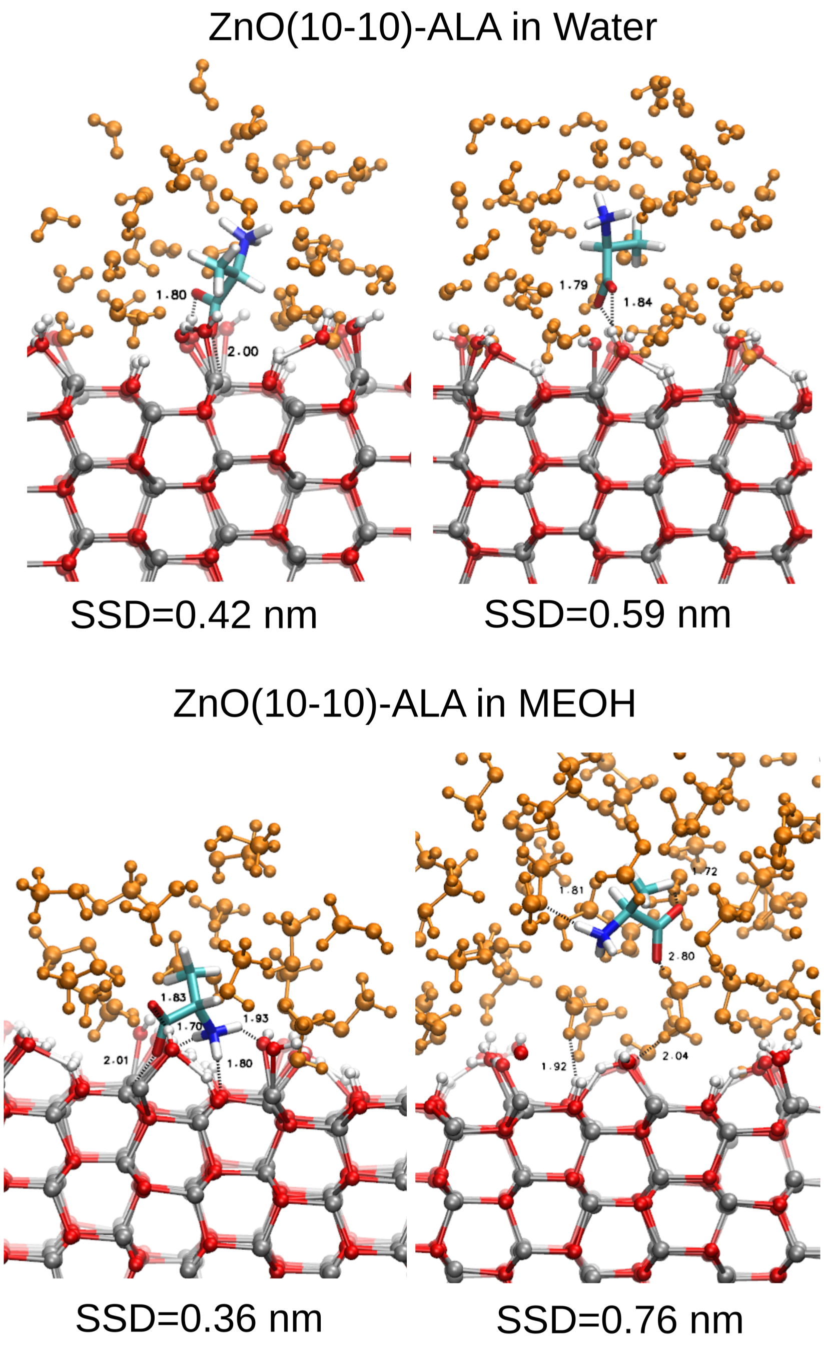

Figure S7: Molecular orientation of ALA adsorption on ZnO(10$\bar{1}$0) and ZnO(10$\bar{1}$0) in presence of water and methanol solvents. Pictures were taken from the side view at two main binding modes. Solvents are shown in orange and some distance to the nearest neighbors are shown in black.
